# Floating Droplet Array: An Ultrahigh-Throughput Device for Droplet Trapping, Real-time Analysis and Recovery

**DOI:** 10.3390/mi6101431

**Published:** 2015-09-30

**Authors:** Louai Labanieh, Thi N. Nguyen, Weian Zhao, Dong-Ku Kang

**Affiliations:** Department of Pharmaceutical Sciences, Department of Biomedical Engineering, Sue and Bill Gross Stem Cell Research Center, Chao Family Comprehensive Cancer Center, Edwards Lifesciences Center for Advance Cardiovascular Technology, University of California–Irvine, 845 Health Sciences Road, Irvine, CA 92697, USA

**Keywords:** floating droplet array, droplet-based microfluidics, droplet trapping, high-throughput analysis, digital detection, droplet clustering

## Abstract

We describe the design, fabrication and use of a dual-layered microfluidic device for ultrahigh-throughput droplet trapping, analysis, and recovery using droplet buoyancy. To demonstrate the utility of this device for digital quantification of analytes, we quantify the number of droplets, which contain a β-galactosidase-conjugated bead among more than 100,000 immobilized droplets. In addition, we demonstrate that this device can be used for droplet clustering and real-time analysis by clustering several droplets together into microwells and monitoring diffusion of fluorescein, a product of the enzymatic reaction of β-galactosidase and its fluorogenic substrate FDG, between droplets.

## 1. Introduction

High-throughput analysis has become an important tool in biology and medicine for elucidating complex biological mechanisms, screening for therapeutic agents, and early diagnosis of disease [1]. These challenging endeavors often require detection of rare biomarkers, such as nucleic acids, proteins, and cells that exist in low abundance among an overwhelming background of interfering species. Moreover, it is often necessary for this analysis to be performed in real-time in order to reveal the dynamic behavior of biological and biochemical processes. Thus, technologies that can isolate, detect, and quantify individual components of a heterogeneous mixture in a highly parallel fashion are needed to meet these challenges. Conventional high-throughput platforms such as high-density microwell plates with robotic dispensing systems have been developed and widely used for high-throughput analysis, such as drug screening. However, they require expensive and bulky robotic machinery and suffer from sample evaporation and comparably large reaction volumes, which can waste precious biological samples or reagents [1,2].

Recently, microfabricated devices have emerged as a powerful experimental platform for performing a diverse range of biological and chemical assays in a high-throughput manner [3,4]. These technologies often permit high-throughput analysis of a complex sample by partitioning a bulk solution into many isolated pico to nanoliter-sized compartments, or microreactors. This discretization confines rare analytes into a small volume, thereby increasing their effective concentration, all the while reducing interference from non-target species [5]. Such partitioning has been accomplished using microfabricated wells [6,7] or chambers in a microfluidic device that are sequestered using pneumatically controlled valves [8]. However, post-analysis retrieval of individual samples is difficult to achieve. Furthermore, mixing of reagents in these devices either requires complex architecture [9] or is often done in bulk before compartmentalization, which may prevent initial reaction products from co-localizing with their initiating target [10].

An alternative approach is to compartmentalize reactions into discrete micron-sized droplets surrounded by an immiscible carrier fluid. Droplet-based microfluidics provides precise control over mixing of fluids, minimizes waste of precious reagents, and reduces evaporation and adsorption of molecules at the device walls [1,11,12]. Monodisperse droplets can be formed at kHz frequencies with sizes precisely controlled by device geometry and fluid flow rates [13]. These droplets can be fused [14–17], split [11], cooled [18], heated [19], and sorted [20,21] on- or off-chip as the application requires. This powerful approach has found many applications such as micro-material fabrication [22], directed evolution [23], mRNA profiling of a heterogeneous population of cells [24], pathogen detection [25], and single-cell [26–28] and single-molecule [5,29,30] analysis.

Hatch and coworkers introduced an ultrahigh-throughput droplet digital PCR (ddPCR) device, whereby tightly packed droplets in a microfluidic chamber were analyzed via an integrated CMOS-based wide-field imaging system for absolute quantification of copy number of target DNA [31]. In this development, they were able to increase the dynamic range of ddPCR 100-fold compared to existing ddPCR systems by increasing the device throughput. However, a small fraction of droplets coalesced, which was likely exacerbated by their tight-packing. Moreover, droplets, which are close together or overlap, complicate image processing and may result in quantification errors in this type of device. Indexing is also a challenge since droplets are free to move throughout the experiment, which hinders real-time monitoring.

Immobilizing droplets into static, spatially-defined arrays facilitates indexing and monitoring of droplets over time since the array element locations create a natural positioning system [1]. Huebner and colleagues used a flow trap to immobilize droplets into a 384-element array, which allowed them to be monitored over time and subsequently recovered by reversing the flow direction [32]. Similarly, Schmitz and coworkers trapped up to 8000 droplets in a chamber via channels containing many constrictions [33]. Droplet recovery was achieved by increasing the flow rate through the chamber. However, both of these methods are not amenable for ultrahigh-throughput analysis because the traps are situated within the flow stream and create resistance to flow at high densities of droplets [33]. Moreover, the majority of the droplets pass around the traps and could potentially waste expensive reagents or samples [32]. It has also been previously reported that droplets in a multilayered device could be trapped by buoyancy forces between the drops and the carrier fluid [34,35]. Despite the utility of such an approach, these devices required precise alignment of the PDMS layers and the highest throughput achieved was only 120 droplet traps, which is comparably low-throughput for many biological applications. Therefore, there is a need to develop new devices that can more precisely control droplet trapping in an efficient, ultrahigh-throughput manner.

**Figure 1:**
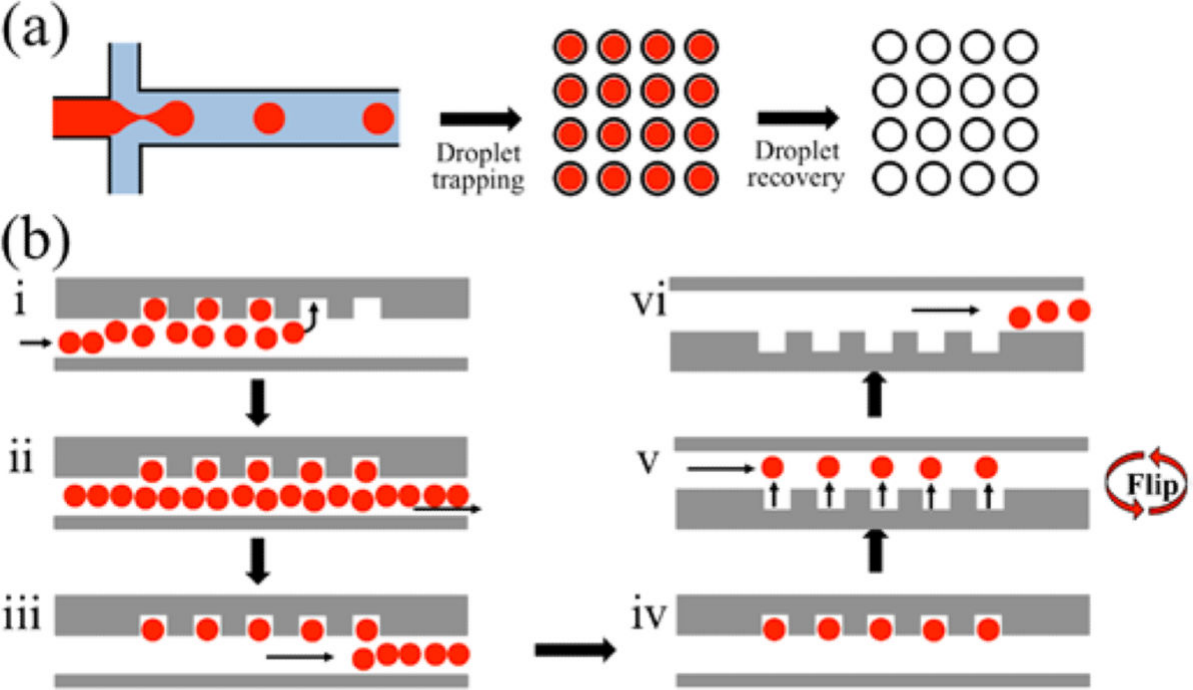
A schematic illustration for the workflow of the Floating Droplet Array (FDA). (**a**) General workflow involves droplet generation, trapping for analysis, and subsequent droplet recovery. (**b**) Step-by-step operation: (i) generated droplets flow into the trapping chamber and float into the wells. After all the wells have been filled (ii), the remaining droplets are purged (iii) and the trapped droplets are then analyzed (iv). Droplets are recovered by flipping the device so that droplets float out of the wells (v) and are sent for downstream handling on- or off-chip (vi).

With the above-mentioned advances and challenges in mind, we have developed a simple and robust dual-layered Floating Droplet Array (FDA) device for ultrahigh-throughput droplet manipulation, analysis, and recovery (Figure 1). The device utilizes density differences between the continuous and discrete phases to trap floating droplets into hundreds of thousands of wells for real-time analysis. Since the droplets are trapped in a secondary layer above the main flow stream, we are able to achieve high density and efficiency of immobilization for real-time, ultrahigh-throughput droplet analysis.

## 2. Experimental Section

### 2.1. Materials

Silicone elastomer base and curing agent (Sylgard 184; polydimethylsiloxane (PDMS)) and HFE-7500 fluorocarbon oil were purchased from Down Corning (Wiesbaden, Germany). Four-inch silicon wafers were purchased from IDB Technologies (Wiltshire, UK). The amphiphilic tri-block copolymer, polyethylene glycol–perfluoropolyether (PFPE-PEG-PFPE), surfactant was synthesized as described below. SU-8 permanent epoxy negative photoresist (SU-8 50) and SU-8 developer were purchased from MicroChem Corp. (Westborough, MA, USA). Streptavidin-modified beads (7.8 μm, COMPEL™ Magnetic) were purchased from Bangs Laboratories (Fishers, IN, USA). Biotin-conjugated β-galactosidase (β-gal) and its fluorogenic substrate (Fluorescein di(β-D-galactopyranoside)) (FDG) were purchased from Sigma-Aldrich (St. Louis, MO, USA). An inverted fluorescence microscope (Eclipse Ti, Nikon, Tokyo, Japan) was used for imaging droplets. An oxygen plasma cleaner (PDC-32G, Harrick Plasma, Ithaca, NY, USA) was used for plasma bonding of the PDMS-PDMS devices. PHD Ultra Syringe Pumps were purchased from Harvard Apparatus (Holliston, MA, USA) and used to control sample injection with syringes.

### 2.2. Device Fabrication Process

The microfluidic device was designed using AutoCAD (Autodesk, San Rafael, CA, USA) and printed to high-resolution transparency photomasks (CAD/Art Services, Bandon, OR, USA). The devices were fabricated from PDMS using standard soft lithography techniques [36]. Four inch silicon wafers were briefly rinsed with 5% hydrofluoric acid (Sigma-Aldrich, St. Louis, MO, USA) and deionized (DI) water. Prior to spin coating (6NPP-LITE, Laurell Technologies Corporation, North Wales, PA, USA), wafers were dehydrated in an oven at 95 °C for 10 min. Negative photoresist (~3 g, SU-8 50, MicroChem, Chestech, UK) was then spin-coated (500 rpm for 10 s then 3000 rpm for 30 s) onto the wafer. The SU-8 layer was then cured on a hotplate at 65 °C for 5 min and at 95 °C for 30 min. The cured SU-8 layer was then exposed to UV radiation (14 s, 20 mW/cm^2^, AB&M INC UV Flood Exposure System) through the photomask and the wafer was subsequently post-baked at 65 °C for 1 min and 95 °C for 5 min. Unexposed SU-8 was removed by soaking in SU-8 developer for 5 min. The wafer was then cleaned using isopropyl alcohol, blow-dried with filtered nitrogen gas, and silanized with perfluorooctyl-trichlorosilane (Sigma-Aldrich, St. Louis, MO, USA) under vacuum for 3 h. For fabrication of the devices, PDMS base and curing agent were mixed in a ratio of 10:1 *w*/*w*, degassed, poured onto SU8-on-Si wafer masters and fully cured overnight in an oven at 65 °C. After thermal curing, the PDMS layer was peeled off the master. Inlet and outlet holes were made with a 1-mm-sized biopsy punch (Kay Industries Co., Tokyo, Japan). PDMS layers were bonded immediately following oxygen plasma treatment and stored overnight before use.

### 2.3. Synthesis of PFPE-PEG-PFPE Surfactant

The biocompatible fluorinated surfactant was synthesized as described by Chen *et al*. [37]. Krytox 157FS(H) (50 g, MW: ~5000 g/mol, Dupont, Wilmington, DE, USA) was dissolved in 50 mL anhydrous HFE-7500 (Sigma-Aldrich, St. Louis, MO, USA) and mixed with excess oxalyl chloride (12.5 g, Sigma-Aldrich). The reaction mixture was then left stirring overnight at 85 °C under an argon atmosphere. A light yellow product was obtained after solvent removal by rotary evaporation and high vacuum. This product was then mixed with Jeffamine XTJ 501 (3.5 g, MW: 900 g/mol, Sigma-Aldrich) and dissolved in a solvent mixture consisting of HFE 7500 (50 mL) and anhydrous dichloromethane (50 mL, Sigma-Aldrich). The reaction mixture was heated to 65 °C and stirred for 2 days under an argon atmosphere, resulting in a milky white product. After solvent removal by rotary evaporation, the product was centrifuged at 8000 rpm for ~10 min to remove white particles. The product was then dried in a vacuum desiccator for 24 h and used without further purification.

### 2.4. Droplet Generation and Manipulation

HFE-7500 fluorocarbon oil (Dow Corning, Midland, MI, USA) containing 1.8% (*w*/*w*) PFPE-PEGPFPE surfactant was used as the continuous phase for droplet generation, and the same oil without surfactant was used for droplet purging. Aqueous and oil phases were injected into the microfluidic device via pressure equalization tubes (Smith Medical, St. Paul, MI, USA). In all microfluidic experiments, PHD Ultra Syringe Pumps were used to inject fluids at flow rates ranging from 0.031 to 30 μL/min. To evaluate the performance of the Floating Droplet Array, 10% red food coloring dye (McCormick, Sparks, MD, USA) was used as the aqueous phase to visualize droplets. The dye was injected via the two aqueous inlets at flow rates ranging from 0.031 to 12 μL/min, whereas the oil phase was injected at flow rates ranging from 10 to 15 μL/min (flow rate was varied to manipulate the size of droplets). Monodisperse droplets were generated by the flow-focusing structure at sizes ranging from 19 to 145 μm in diameter by tuning the microfluidic channel size and fluid flow rates. Large area scans of trapped droplets were imaged in stitching mode using a 4× objective lens and a fluorescence microscope (Eclipse Ti, Nikon, Tokyo, Japan).

### 2.5. Preparation of β-Gal-Conjugated Beads (β-Gal Beads)

For the preparation of β-gal conjugated beads, streptavidin-modified beads (1.8 × 10^7^ beads, 7.8 μm) were incubated with biotinylated β-gal (1.4 mM, 100 μL) for 60 min at room temperature. The solution was mixed by pipetting every 10 min to prevent settling of the beads. After incubation, beads were washed ten times with 100 μL of phosphate-buffered saline (PBS, 137 mM NaCl, 2.7 mM KCl, 10 mM Na_2_HPO_4_, 2 mM KH_2_PO_4_, 10 mM MgCl_2_ pH 7.4) to remove unbound β-gal.

### 2.6. Fluorophore Diffusion between Droplets

For fluorophore diffusion studies, β-gal beads and 500 μM FDG in PBS were introduced into the microfluidic device via respective inlets at a flow rate of 0.5 μL/min, while the oil phase was injected at a flow rate of 15 μL/min. A 2-mm magnetic stir bar was placed inside a 3 mL syringe and was gently mixed by a portable magnetic stirrer (Utah Biodiesel Supply, Syracuse, UT, USA) to prevent settling of the beads. Uniform 55 μm diameter droplets were generated, such that three droplets could fit within 120 μm diameter microwells. The droplets were incubated at room temperature and the fluorescence intensity of droplets and surrounding oil phase was analyzed under a fluorescence microscope at various time points to monitor the fluorophore-leaking effect between droplets.

### 2.7. Digital Quantification of β-Gal Beads

For the digital quantification of β-gal beads using the FDA device, 25 μm sized-droplets, containing 250 μM FDG with or without a single β-gal bead were trapped within the microfluidic device consisting of 109,569 microwells (30 μm in diameter). After a 10-min incubation, microscopic images were taken using a 4× objective lens. The experiments were performed in triplicate and the resulting images were analyzed using ImageJ software (ver. 1.48, http://imagej.nih.gov/ij/) for quantification of fluorescent droplets.

## 3. Results and Discussion

### 3.1. Design of the FDA Device for Ultrahigh-Throughput Droplet Trapping

**Figure 2:**
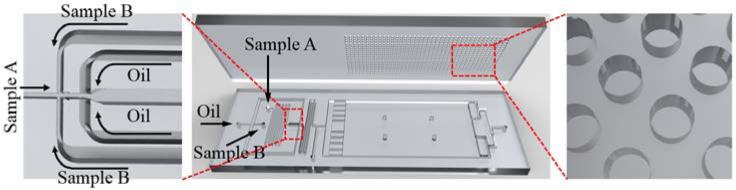
CAD rendering of the Floating Droplet Array. The top layer of the FDA device contains the droplet-trapping microwells while the bottom layer contains the droplet generation and chamber modules (**Middle**). Droplets are generated using a flow-focusing structure (**Left**) and trapped into circular microwells (**Right**). Device geometries were exaggerated in the rendering for visualization purposes.

**Figure 3:**
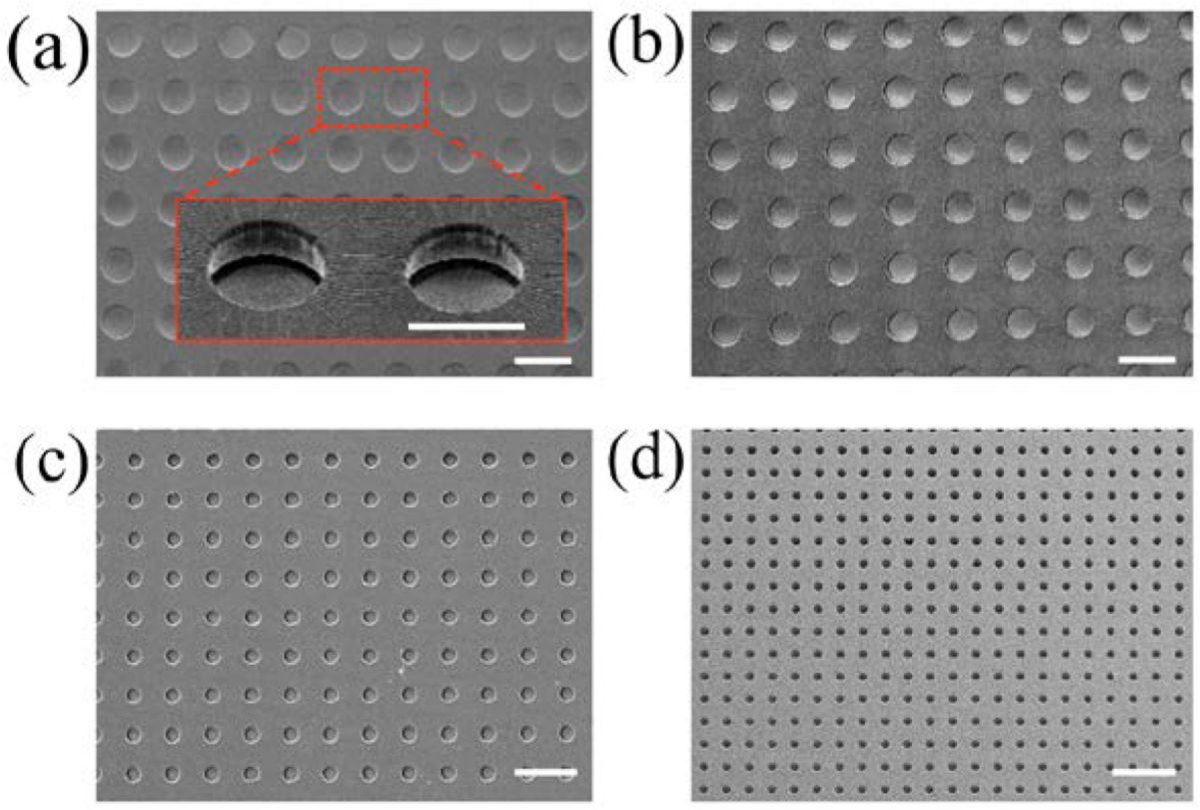
SEM images of the microwells. Top view images of (**a**) 120 μm diameter wells (8457 total); (**b**) 100 μm wells (13,232 total); (**c**) 50 μm wells (34,344 total); and (**d**) 30 μm wells (109,569 total); Inserted image in (a) was taken at 45°. Scale bar = 200 μm for (a–d) and 120 μm for insert in (a).

**Figure 4:**
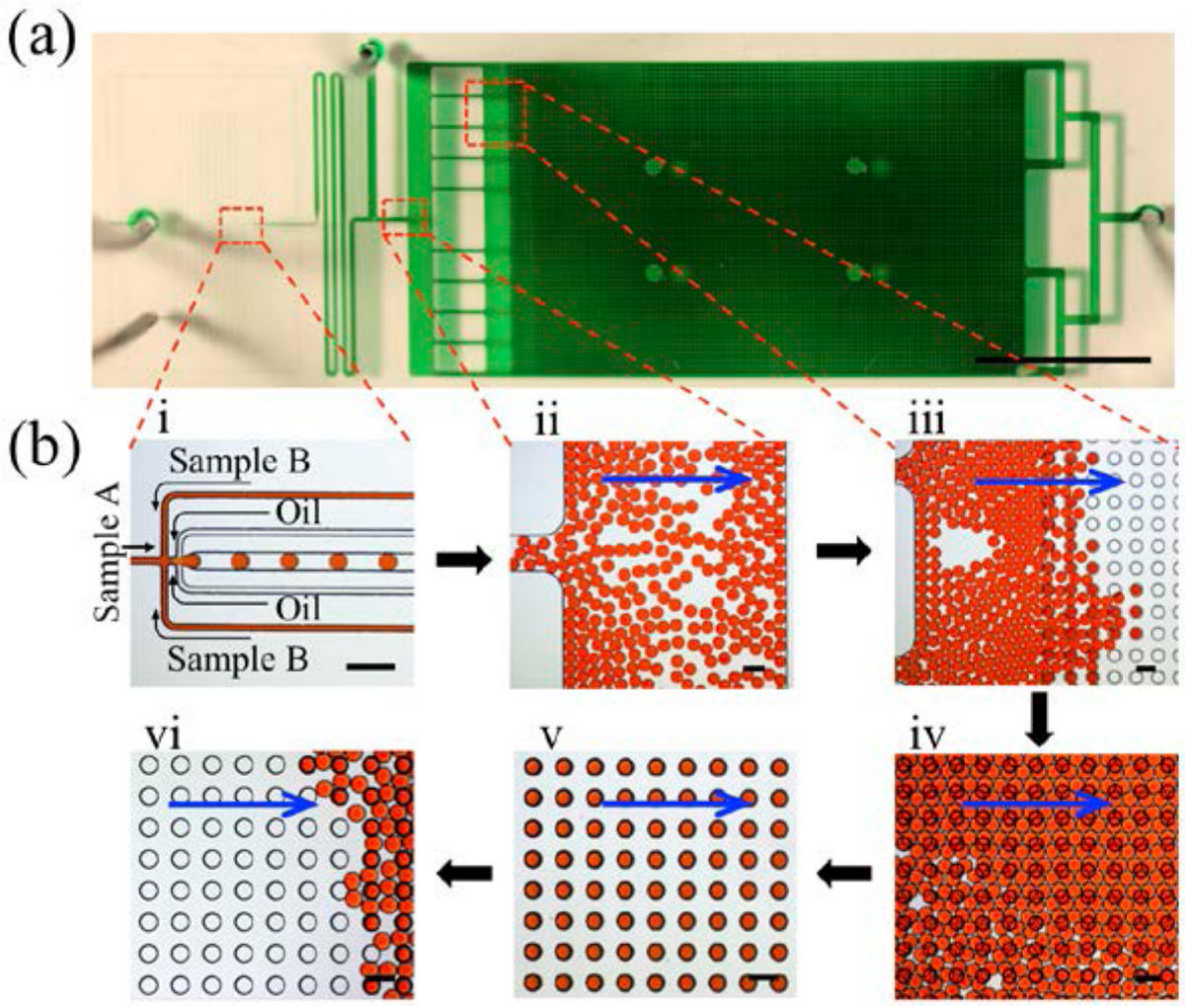
Images depicting the workflow for the Floating Droplet Array. (**a**) Photographic image of entire microfluidic device. The device was filled with green dye for visualization, scale bar = 1 cm; (**b**) Microscopic images of the workflow including (i) droplet generation, (ii) droplet loading into the chamber, (iii) droplet trapping, (iv) filling the chamber, (v) purging extraneous droplets, and (vi) droplet recovery by flipping. Blue arrows in (ii–vi) represent flow direction. All scale bars for (b) = 200 μm.

A rendering of the FDA device design is shown in Figure 2. The FDA device consists of two layers of PDMS, one for droplet generation and assembly and the other for droplet trapping. The top layer is designed with a microwell array whose well dimensions can be varied according to the desired droplet size to be trapped. In this work, we used the dimensions (well width × depth) of 30 × 40, 50 × 50, 100 × 50, and 120 × 50 μm (Figure 3). Fabricated microwells in the top PDMS layer were characterized by scanning electron microscopy (SEM) as shown in Figure 3. The diameter of microwells were determined to be 122.5 ± 6.1, 96.7 ± 4.7, 48.6 ± 2.3, and 27.8 ± 1.4 μm, which correspond to a total well number of 8,457, 13,232, 34,344 and 109,569, respectively. The bottom PDMS layer was fabricated with a height of 50 μm and contains two aqueous inlets and a single oil inlet whereby the respective fluids are directed to a flow-focusing structure for droplet generation (middle panel, Figure 2). The channel width at the flow-focusing structure is 15 μm when the 30 or 50 μm diameter wells were used and 30 μm when the 100 or 120 μm diameter wells were used. After the flow-focusing structure, we included a widened winding channel, which reduces the velocity of the droplets and aides in droplet visualization. The bottom layer also contains a large chamber (18.5 mm wide × 37 mm long), which is oriented below the well array. We placed nine large rectangular-shaped resistor structures with long and narrow channels (3 mm long, 200 or 300 μm wide) between them immediately after the entrance of the chamber (Figures 2 and 4a). This provides resistance to flow down the length of the chamber and ensures that droplets spread out across the whole width of the chamber before passing through the narrow channels to the well array (Figures 2 and 4a). We found this helps to ensure compete coverage of the wells. The chamber also contains four pillar structures (1 mm diameter) placed in the central region of the chamber to prevent undesirable bonding of the well array with the bottom of the chamber due to bowing of the PDMS (Figures 2 and 4a). The outlet channels (550 μm wide) are designed at the end of the chamber for collecting excess oil and also to recover the trapped droplets from the FDA device. We also included a waste outlet before the entrance to the chamber to divert undesired droplets such as air, polydisperse, or improperly-sized droplets which often occur at the beginning of device operation from the microwell array. Once generation of the desired droplet size was stable, this waste channel was sealed with a stopper and the droplets were diverted into the chamber for trapping.

### 3.2. Droplet Generation and Manipulation for the Trapping

Figure 4b shows a step-by-step workflow for the FDA device using dye-containing droplets trapped and released in 120-μm microwells. To operate the device, we initially purged the chamber of air by flowing oil (HFE 7500 without surfactant) through the oil inlet at a flow rate of 10 μL/min for 5 min. Aqueous samples were then introduced for droplet generation with the device oriented so that the wells were above the chamber. We generated droplets using HFE 7500 + 1.8% PFPE-PEG-PFPE surfactant as the oil phase and 10% food coloring dye as the aqueous phase for generating droplet sizes ranging from 20 to 120 μm in diameter by varying the oil and aqueous flow rates (i in Figure 4b). Initial droplets were diverted into the intermediate waste outlet until the desired droplet size was stably formed. The waste outlet was then sealed with a stopper and the droplets were consequently guided into the chamber, where they spread across the width of the chamber before passing through the narrow channels between the resistor structures (ii in Figure 4b). The droplets then sequentially filled the wells by floatation due to the density difference between the fluorinated oil and aqueous phase (iii in Figure 4b, and Movie S1). Once the array was completely filled (iv in Figure 4b), the aqueous inlets were sealed and oil was introduced at a high flow rate (20–30 μL/min) for 10 min to purge the chamber of any residual droplets (Movie S2). The trapped droplets were then incubated and analyzed over time (v in Figure 4b).

**Figure 5:**
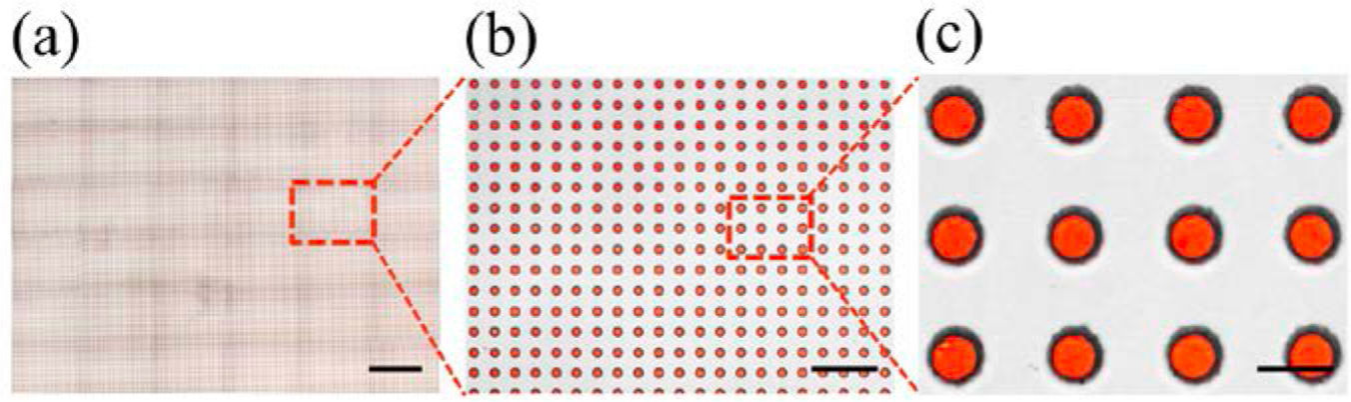
Microscopic images of the ultrahigh-throughput FDA using 50 μm wells. (**a**) Large-scale scan of 36 images containing more than 14,000 wells and (**b,c**) zoomed-in images of trapped droplets.

Subsequently, the droplets were recovered by flipping the device over and applying a fast flow rate of oil so that the droplets float out of the wells and are collected off-chip (vi in Figure 4b and Movie S3). This simple technique is robust and can be applied to a wide range of droplet sizes. Moreover, it is highly efficient in trapping droplets as can be seen in Figure 5 with 100% of >14,000 wells analyzed containing a single droplet. We found that with the device dimensions used in this study, we can consistently fill nearly 100% of the microwells with single droplets when they are generated to be 10%–20% smaller in diameter compared to the microwells (Figure 5a and Movie S4). In our studies, we used HFE 7500 and 1.8% PFPE-PEG-PFPE surfactant as the oil phase because of the biocompatibility and droplet stability of this oil-surfactant system. The resulting aqueous droplets float into the wells due to the higher density of the fluorinated oil relative to the aqueous phase. However, we anticipate that this device can also be used in emulsions where the droplets are trapped by sinking into the wells.

**Figure 6:**
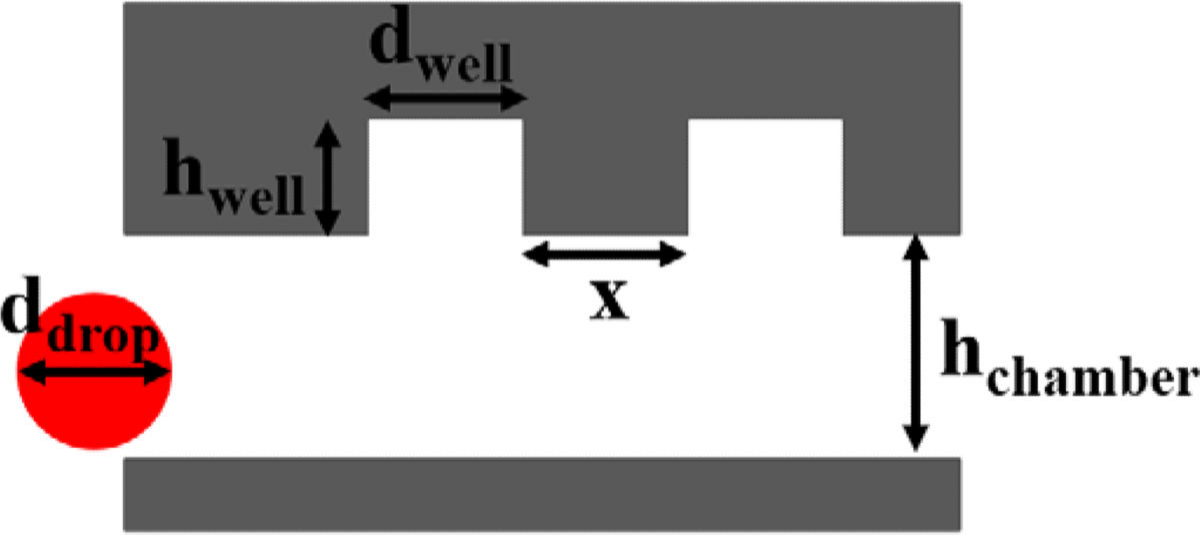
Device design parameters for efficient droplet trapping and recovery.

Geometric parameters of the device such as the diameter of the well, *d*_well_, depth of the well, *h*_well_, height of the chamber, *h*_chamber_, and inter-well spacing, *x*, must be chosen accordingly to efficiently trap and release a droplet in a well (Figure 6). The chamber height must be sufficient enough not to cause clogging as the tightly-packed droplets pass through the chamber. Droplets with diameters much greater than *h*_chamber_ (*i.e.*, >3x) appear flattened and are trapped into the wells as they attempt to minimize their surface energy [38,39]. However, these oversized droplets cause clogging of the device at localized regions, which create “dead zones” of low flow. We found it difficult to process such droplets in the large, high-density array format of the FDA. Conversely, if the height of the chamber is more than two times the diameter of the droplet, then the droplets are not efficiently trapped by floatation into the wells under high oil flow rates (>7 μL/min) and flow must be reduced to efficiently trap the droplets. Moreover, if the depth of the well is too deep, then multiple droplets may be trapped within each well. For geometries of *d*_well_ = 50 μm, *h*_well_ = 50 μm, *h*_chamber_ = 50 μm, and *x* = 75 μm, we found that a droplet diameter of 40–50 μm was optimal for efficient entrapment and release. Generally, we found that the ideal parametric values are related as *d*_well_ = *h*_well_ = *h*_chamber_ = 1.17 *d*_drop_ and *x* from 40 to 75 μm for efficient droplet trapping and recovery. This was determined experimentally for droplets in the range of 30 to 100 μm. However, there is considerably less restraint placed on these parameters if only droplet trapping is desired. As seen in Movie S4 and tabulated in Table 1, the optimized parameters result in near complete coverage, with greater than 99% of wells containing a single droplet. Moreover, droplet recovery was efficiently achieved by reorienting the device and applying a high oil flow rate. The ability to recover individual droplets is a subject of future work, but we anticipate that this may be achieved using microneedle [40], valve [41], electrode [42], or laser-based techniques [43]. The time required to trap droplets depends on the droplet size, but we found that we can typically fill the wells within 5–10 min, with an additional 10 min needed to purge the extraneous droplets. Reducing the washing time could be achieved by integrating additional oil inlet and outlet channels so that droplets are washed across the width of the chamber. The capture efficiency, which is the percentage of trapped droplets relative to the total number of generated droplets, ranged from 14.8% to 22.8%. However, this number could be improved for applications using precious reagents by terminating droplet generation prior to the droplets completely filling the chamber. For example, droplet generation can be ceased when the droplets have covered 50% of the wells and allowing the remaining free-floating droplets to fill the empty wells. Although this might result in a slight decrease in the coverage of the array.

**Figure 7:**
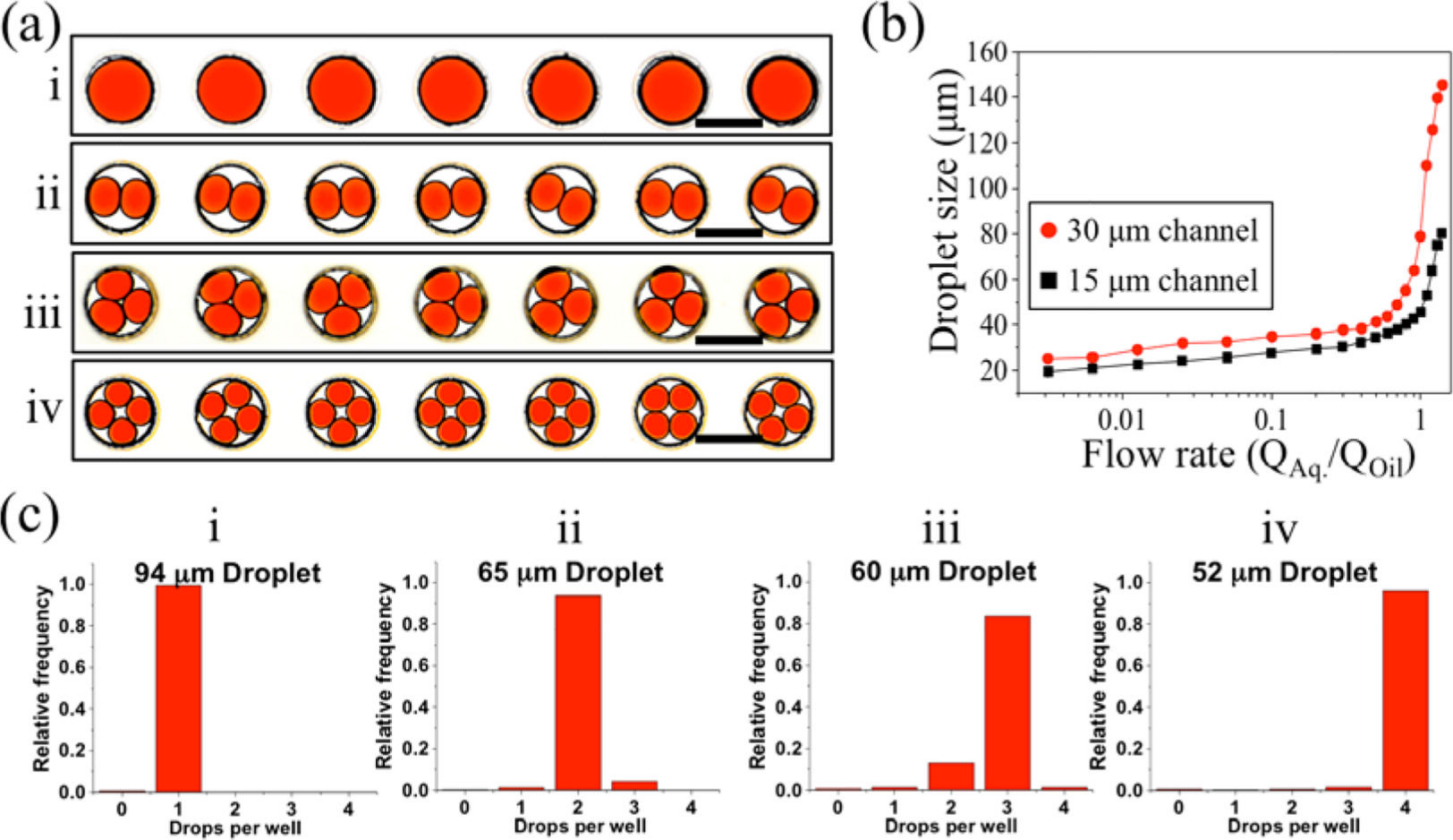
Controlling the number of droplets per well. (**a**) Microscopic image showing multi-droplet clustering in 120 μm wells; one droplet per well (i), two droplets per well (ii), three droplets per well (iii) and four droplets per well (**iv**). Scale bars =120 μm. (**b**) Controlling droplet size by manipulating the flow rate ratio (water/oil) for 30 × 50 μm channel (red) and 15 × 50 μm channel (black). (**c**) Distribution of droplet occupancy in 120 μm wells for droplets with a diameter of (i) 94, (ii) 65, (iii) 60, and (iv) 52 μm. Occupancy was quantified based on analysis of at least 432 wells for each group.

As a further demonstration of the versatility of this device for droplet trapping, we clustered multiple droplets into a single well in a simple, robust, and well-controlled manner. This was achieved by varying the size of the droplets so that more than one droplet could fit into each well. As seen in Figure 7a, we were able to precisely manipulate one, two, three, and four droplets per well by controlling the droplet size. Droplet size was experimentally optimized for controlling multi-droplet clustering in the wells (Figure 7b). With optimized droplet-sizes (94, 65, 60 and 52 μm), the efficiencies of single, double, triple and quadruple droplet trapping were determined as 99.27%, 93.62%, 83.70%, and 96.11%, respectively (Figure 7c). This ability of the FDA device can be used for clustering multiple droplets that contain different samples or reagents within the same microwell for various complex biological studies such as enzymatic assays, drug screening, and cell-cell communication. This may be achieved by controlling diffusion (crosstalk) of molecules between droplets or merging droplets within the same microwell, in a highly parallel manner.

### 3.3. Identification of Crosstalk between Droplets

**Figure 8:**
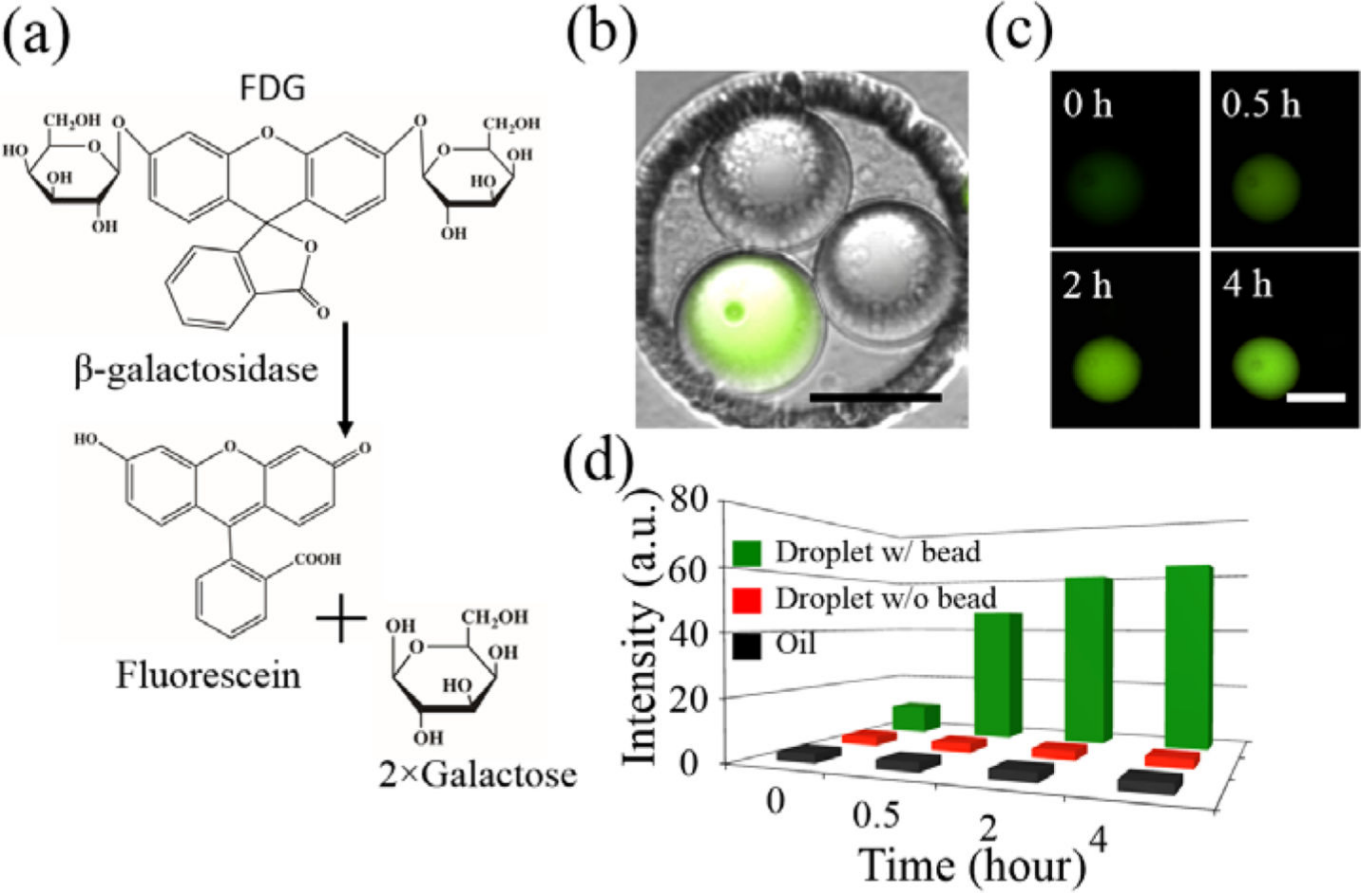
Droplet cross-talk studies of the diffusion of fluorescein between clustered droplets. (**a**) Hydrolysis of FDG by β-gal. (**b**) Microscopic image showing overlay of bright-field and FITC channels for FDG droplets with and without a β-gal bead after a 4-h incubation, scale bar = 50 μm. (**c**) Fluorescence microscopic images showing time course of reaction over a 4-h incubation to monitor droplet crosstalk, scale bar = 50 μm. (**d**) Fluorescence intensity of trapped droplets with bead (green), without bead (orange), and the oil phase (black).

It has been previously reported by several groups that some small hydrophobic molecules such as fluorophores can diffuse between droplets, which can interfere with biological assays such as enzymatic activity assays and single cell studies [44–50]. To investigate this phenomenon in our FDA device, we chose β-gal and its fluorogenic substrate (FDG) as a model system (Figure 8a) because the fluorescent product of this reaction, fluorescein, has been reported to leak between droplets [47]. We encapsulated a dilute solution of β-gal beads (500 beads/μL) with 250 μM FDG which resulted in only a few droplets containing a β-gal bead and most droplets containing FDG without any β-gal beads. We controlled the droplet size to be 55 μm such that 3 droplets were trapped in most of the wells (Figure 8b). We then monitored the fluorescence of the β-gal bead-containing droplets, neighboring blank droplets, and the surrounding oil over time (Figure 8c). However, with the oil phase and surfactant used in this study, we were not able to observe any diffusion of the fluorophore into the neighboring droplets over the 4 h incubation period (Figure 8d). We confirmed that the droplets were making good contact with one another but could not observe any fluorescence signal diffusing into neighboring droplets, even after a 12-h incubation (data not shown). We believe that the discrepancy between our results and a previously reported study [47] is due to the alternative oil phase we used in our experiments (HFE-7500 fluorocarbon oil containing 1.8% (*w*/*w*) PFPE-PEG-PFPE surfactant), which is known to have superior droplet stabilization compared to other oil-surfactant systems [51]. This finding is consistent with previous reports that studied fluorescein transport between droplets using fluorinated oils and PEG-PFPE-based surfactants [45,50]. It is possible, however, that fluorescein could be transported through the oil phase, as has previously been shown to occur with 4-methylumbelliferone using mineral oil and Abil EM90 surfactant [48]. However, we believe this effect must have been negligible in our experimental setting since we did not observe a decrease in signal intensity from the fluorescent droplets or an increase in the background fluorescence.

For prolonged incubation periods, we observed shrinkage of the droplets due to evaporation of the aqueous phase through the PDMS (29.6% reduction in volume after 5 h, Figure S1) [52]. Moreover, the droplets deformed and appeared flattened in the wells after 6 h. This deformation was observed to propagate in a spatially-dependent manner, which suggests that it was due to the PDMS device itself. This phenomenon could likely be attributed to swelling and pressure-induced deformation of the PDMS. The use of other materials that do not suffer from these permeability and deformation effects, such as glass or poly(methyl methacrylate) might be appropriate for applications requiring long-term incubations [53].

We believe that there is great potential in using the FDA platform to cluster multiple droplets of differing contents (*i.e.*, cells, reagents, or samples) and merging them using a chemical reagent [16] or externally applied electric field [17]. This has been previously achieved in channels using a sequential format where droplets were merged in series and also required that they be synchronized in some manner to bring together, typically, pairs of droplets [16,17,54]. However, with the FDA device, one can colocalize and merge multiple droplets per well in a highly parallel and controlled fashion, which also carries the advantage of merging thousands to millions of droplets simultaneously and can be used for various complex biological experiments such as single-cell communication studies, enzymatic assays, drug screening, *etc.*

### 3.4. Digital Quantification of Single β-Gal Beads

**Figure 9:**
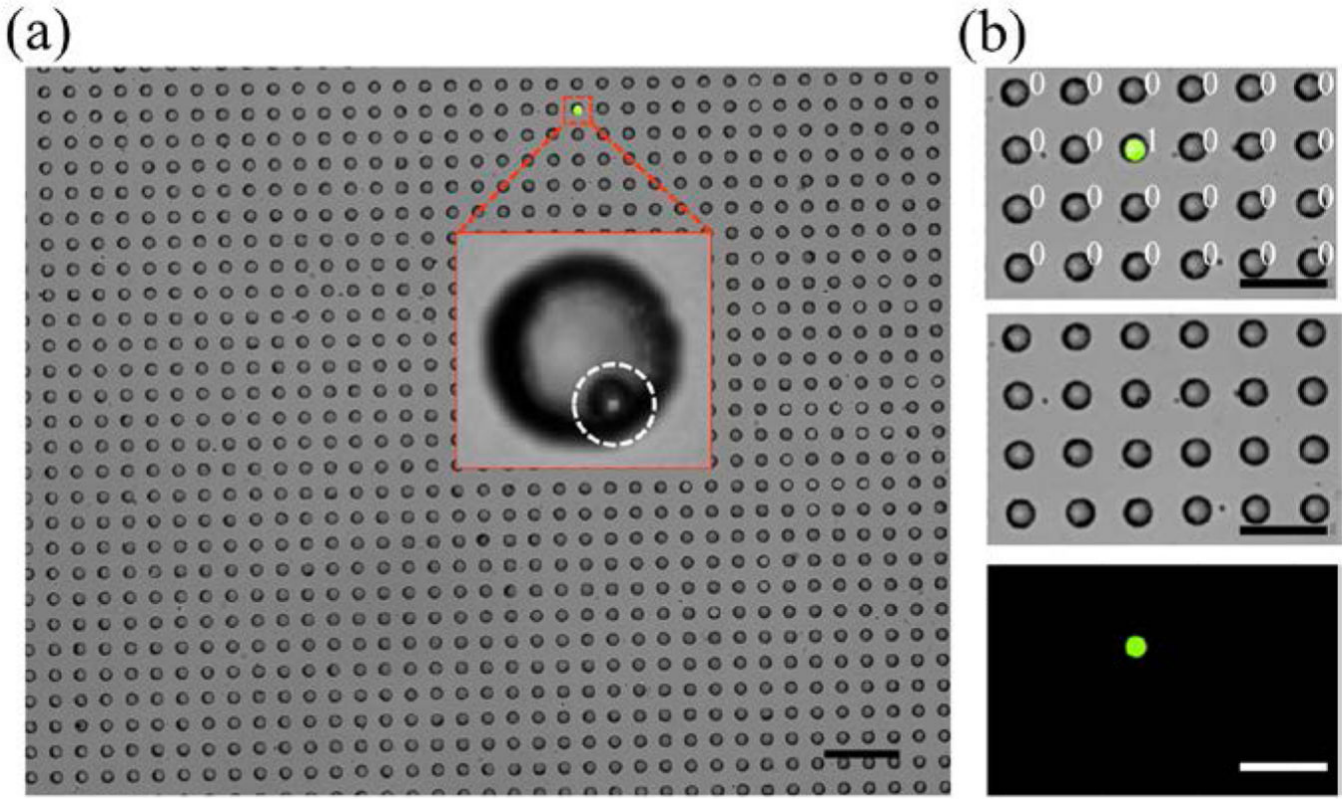
Digital quantification of the number of droplets containing β-gal bead in the FDA device. (**a**) Microscopic image of trapped droplets in a device containing 109,569 microwells of 30 μm size. Droplets are generated with 250 μM FDG and a low concentration of β-gal beads so that most droplets do not contain any beads. Insert depicts a zoomed bright field microscopic image of a bead-containing droplet. White circle highlights a β-gal bead (7.8 μm) within a droplet. (**b**) Fluorescence microscopic images of droplets within 30 μm microwells. 0 = without target bead (dark droplet without β-gal bead), 1 = with bead (fluorescent droplet with β-gal bead). All scale bars = 200 μm.

Recently, droplet-based microfluidic technologies have been showing great promise in digital-based absolute quantification of rare biological molecules using droplet digital PCR [54,55], droplet digital ELISA [56], and digital single-cell quantification/detection [5,25–27]. However, one of the biggest challenges has been the difficulty in indexing a large number of droplets for real-time monitoring. To demonstrate the potential of our FDA device for digital quantification with indexed droplets, we encapsulated FDG along with a very low concentration of β-gal beads (10 beads/μL) so that the majority of droplets contain no β-gal bead and only a few droplets contain only one bead. Streptavidin-conjugated beads (7.8 μm) were used since they can be easily visualized and can also immobilize a large number of β-gal molecules, to yield strong enzymatic activity. As can be seen in Figure 9a, there is only one fluorescent droplet, and it is the only one that contains a β-gal bead, among 1008 droplets in the image.

We counted 5.33 ± 2.08 fluorescent droplets, all of which contained a single β-gal bead, while the remaining droplets were not fluorescent, from triplicate experiments. The concentration of the beads in the solution was determined to be 5.33 beads in 0.9 μL (8.2 pL droplets × 109,569 wells) which is in excellent agreement with the original bead concentration (10 beads/μL) before encapsulation into 25 μm droplets. In this demonstrative study, we were able to digitally count the number of single β-gal beads by monitoring fluorescent droplets trapped in the microwells (Figure 9b).

## 4. Conclusions

Here, we have presented a novel, ultrahigh-throughput FDA device for immobilizing more than 100,000 droplets in an array of microwells, but it can easily accommodate 10’ s to 100’ s of millions of wells by increasing the size of the chamber or using a higher density of microwells, and is simple to fabricate and assemble. By trapping droplets in a secondary layer above the main flow stream, we have achieved greater throughput and efficiency of immobilization compared to previously developed droplet microfluidic devices for droplet trapping. Moreover, alignment between the two layers does not need to be precise and can be done manually by eye without any specialized equipment. Furthermore, droplet trapping and recovery are rapidly and passively accomplished by exploiting buoyancy forces through simple reorientation of the device and can be readily coupled to further processing on or off-chip.

This technology combines the advantages of compartmentalizing samples by droplet microfluidics with the ultrahigh-throughput analytic and parallelization capabilities of microarray formats. Moreover, immobilizing droplets in this manner yields facile indexing of droplets that is needed for real-time monitoring over an extended period of time. We have shown that this robust and versatile platform can be used for droplet isolation, clustering, digital quantification, and monitoring reactions over time. We envision that it can be used for many other applications such as single-cell or molecule analysis, genetic sequencing, biochemical profiling, cell culture, pathogen detection, and drug discovery. Our ongoing work involves adapting this device for manipulating (e.g., splitting, fusing, *etc.*) a large number of droplets in a parallel fashion, which up until now has primarily been achieved in sequential formats. We envision that this device has great potential for portable, point-of-care technologies when combined with CMOS, CCD, or cell phone-based imaging systems.

## Supplementary Material

Movie S1

Movie S2

Movie S3

Movie S4

Supplemental

## Figures and Tables

**Table 1:** Performance metrics of the Floating Droplet Array (FDA) device. Device performance was analyzed using four different FDA devices to trap a single droplet per well. Droplet coverage (percentage of occupied wells), droplet recovery (percentage of droplets recovered after trapping), and capture efficiency (percentage of droplets trapped relative to total droplets generated) were quantified based on analysis of >1000 wells for each group.

Well diameter (μm)	Total wells	Droplet size (μm)	Droplet coverage (%)	Droplet recovery (%)	Capture efficiency (%)
30	109,569	25	99.92	97.26	16.97
50	34,344	46	99.87	96.13	14.78
100	13,232	82	99.28	89.04	22.77
120	8,457	94	99.27	90.56	20.96
