# Supplementary material for: Floating Droplet Array: An Ultrahigh-Throughput Device for Droplet Trapping, Real-time Analysis and Recovery
Source: Micromachines (Basel). Author manuscript; Available in PMC 2016 Apr 28. (PMC4849166; doi:10.3390/mi6101431)
Supplement: Supplemental [file NIHMS760085-supplement-Supplemental.pdf]

Supplementary Materials

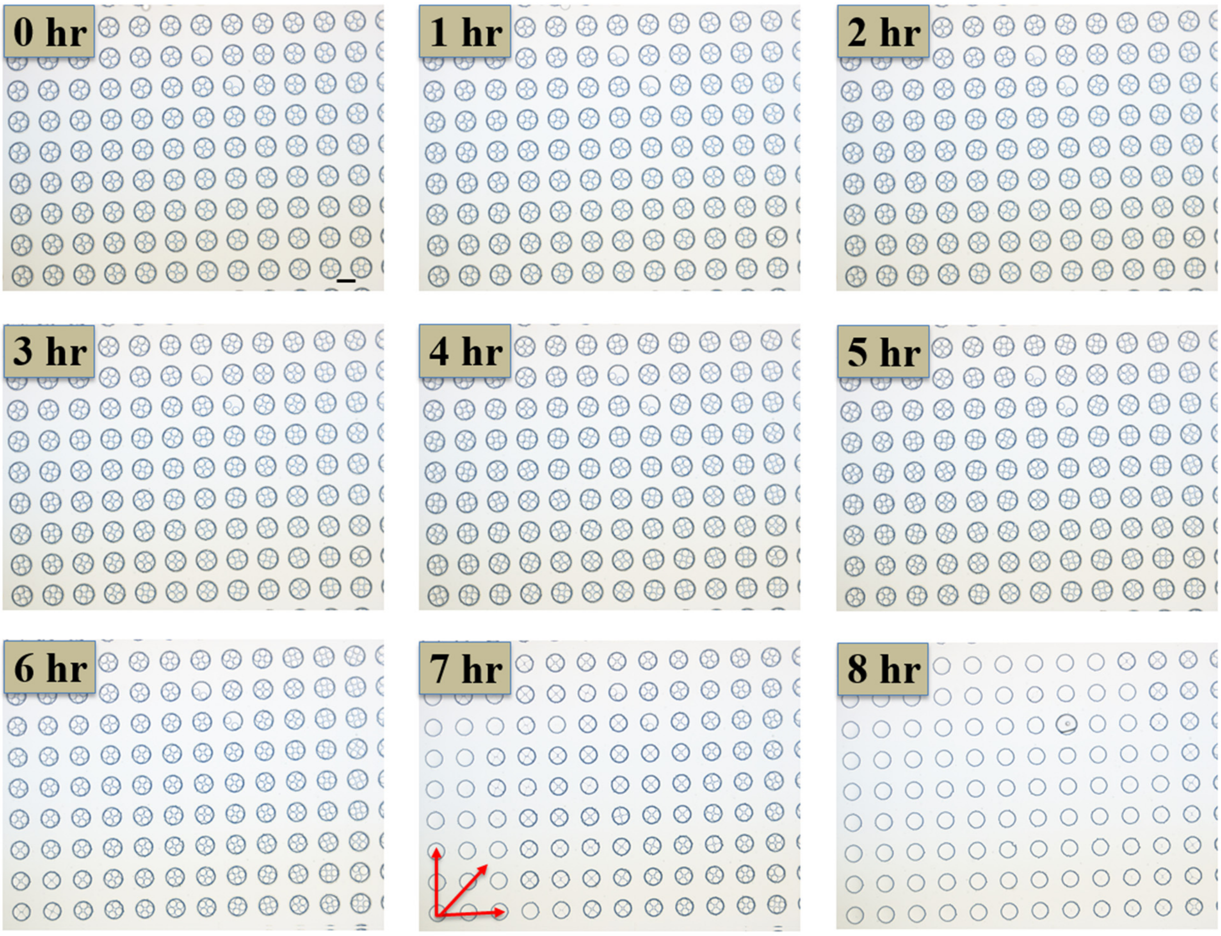

Figure S1. Deformation and evaporation of droplets during extended incubation times. Droplets shrink due to evaporation (29.6% reduction in volume after 5 h, n = 60). After five hours, droplet deformation in the wells propagated from the lower left hand corner of the image to the top right hand corner. The red arrows at 7 h represent the direction of propagation for droplet deformation.

Movie S1. Representative video demonstrating droplet trapping in the Floating Droplet Array (FDA) device. 94 µm-sized droplets were generated and trapped within 120 µm wells.

Movie S2. Representative video demonstrating purging of residual droplets. After droplet trapping, residual droplets were purged with a high oil flow rate (10–20 µL/min).

Movie S3. Representative video demonstrating droplet recovery after flipping and applying a high oil flow rate.

Movie S4. Representative video showing a large-scale scan of trapped droplets. 46 µm-sized droplets were trapped in 50 µm wells and imaged under a microscope.
